# Maternal associated factors of low birth weight: a hospital based cross-sectional mixed study in Tigray, Northern Ethiopia

**DOI:** 10.1186/s12884-015-0658-1

**Published:** 2015-09-17

**Authors:** Meresa Gebremedhin, Fentie Ambaw, Eleni Admassu, Haileselassie Berhane

**Affiliations:** Department of Public Health, Aksum University, College of Health Sciences, P.O. Box-1010, Aksum, Ethiopia; Bahir Dar University, College of Health Sciences, School of Public Health, Bahir Dar, Ethiopia

**Keywords:** Low birth weight, Maternal factors, Tigray, Northern Ethiopia

## Abstract

**Background:**

Birth weight is an important determinant of child survival and development. So far, the prevalence and traditional maternal feeding practice correlates of low birth weight have not been explored well in Ethiopia. Therefore, the purpose of this study was to determine the prevalence and associated factors of low birth weight among mothers who delivered at governmental hospitals, Northern, Ethiopia.

**Methods:**

A cross-sectional mixed study design was carried out in 3 zonal hospitals among 308 mothers and their respective live born baby consecutively using interviewer administered piloted questionnaire and 3 focus group discussions were conducted for the qualitative part. The mothers were interviewed and assessed within 6 hours of delivery; all babies were weighed on standard beam balance from Aug 2 to Sep 12. Data were entered, cleaned and analyzed using SPSS version 16.0. Bivariate and multivariate logistic regression was employed to identify the predictors at *p* < *0.05*. For the qualitative study design, following iterative hearing of the discussions verbatim interpretation was done & categorized in to themes and finally triangulated with the quantitative results.

**Results:**

The prevalence of low birth weight was found to be 14.6 % (95 % CI = 12.56-16.61) and the mean and standard deviations of the birth weights were 3094.9 ± 587.6 grams. Low birth weight was associated with rural place of residence (AOR = 4.34 (95 % CI = 1.99-9.48)), preterm birth/gestational age less than 37 weeks (AOR = 18.5 (95 % CI = 4.94-69.4)), presence of any chronic medical illness (AOR = 5.3 (95 % CI = 1.12-25.45) and maternal weight <50 kg (AOR = 2.26 (95 % CI = 1.06-4.80)). It was found that tradition of selection and preference of nutritionally poor food items during pregnancy was deep-rooted in the community.

**Conclusion:**

The prevalence of low birth weight was found to be high and it was associated with rural place of residence, gestational age <37 wks, presence of any chronic medical illness & maternal weight <50 kg. Emphasis should be given to nutritional counseling and disease specific ANC provision by skilled health professionals; Discussions with the community and religious leaders are mandatory aspect on the tradition of feeding of pregnant mothers to tackle the problem. This study also calls for community based further studies.

## Background

The World health organization (WHO) defined low birth weight (LBW) as weight at birth of less than 2,500 grams. This practical cut-off for international comparison is based on epidemiological observations that infants weighing less than 2,500 grams are approximately 20 times more likely to die than heavier babies [1]. The prevalence of LBW in any population reflects its socio-economic development and it is a good proxy to gauge the developmental status of the country [2].

Low birth weight is associated with many socio-economic factors such as residence (urban-rural difference), mother’s age and occupation, birth order, the family’s income and many maternal conditions such as nutritional status, mother’s educational and health status [3]. Known factors for pre-term delivery and fetal growth retardation which are associated with LBW include low maternal food intake and illness, especially infections. Studies suggest that short maternal stature, very young age, high parity, close birth spacing were all associated factors [4, 5].

More than 20 million infants worldwide, representing 15.5 percent of all births are born with low birth weight, 95.6 percent of them in developing countries. The level of low birth weight in developing countries 16.5 % is more than double the level in developed regions that is 7 % [1, 4].

In Ethiopia different studies have reported the prevalence of LBW from live birth hospital records, the prevalence of low birth weight was around 11 % [6], increasing in pattern a study conducted in Jimma reported that prevalence of low birth weight was 22.5 % [7] and a recent study in Gondar showed 17.1 % [5]. However, all those previous studies were conducted most on the socio-economic factors lacking pertinent variables of socio-cultural factors like feeding of mothers during the course of pregnancy. Therefore this study was intended to include these overlooked factors that were appropriate to this study design. Hence, this study was aimed to fill the knowledge gap of the region concerning the socio-cultural aspects particularly the tradition of feeding of pregnant mothers of the study area.

## Methods

### Study design

Institution based cross-sectional concurrent quantitative-qualitative study design was used to describe the magnitude and associated factors of LBW and the socio-cultural factors were described using qualitative study design.

### Study area and period

Tigray forms the northernmost reaches of Ethiopia, and is located between 36 degrees and 40 degrees East longitude. Its North-South extent spans 12 and half degrees to 15 degrees north. It is bordered by Eritrea in the North, Sudan to the West, Amhara national state to the Southwest and Afar of Ethiopia to the East. Tigray has an estimated population of 4,565,000. The region has health service coverage of 83 %. Central zone is one of the six zones and it is around 1024 KMs away from Addis Ababa, capital of Ethiopia. Has a total population of 1,245,824, of whom 613,797 were men and 632,027 were women; 176,453 or 14.16 % are urban inhabitants [8]. the central zone consists of 3 hospitals: Aksum St. Mary hospital, Adwa hospital and Abi-Adi hospital.

The study was conducted from Aug 2 to Sep 12, 2013.

### Study population and study unit

Mothers/newborn pairs at governmental hospitals of Tigray, Northern, Ethiopia and study units were mothers and their respective newborn found during the data collection in central zone of Tigray.

### Inclusion and exclusion criteria

All mothers that gave birth in the hospitals of central zone were included and mothers suffering from severe medical or surgical condition, twin delivery, mothers with unknown last menstrual period (LMP), and still birth, at the time of enrollment were excluded from the study.

### Sample size calculations

The required sample size was determined using single-population proportion with the following assumption: 22.5 % of prevalence of LBW in Ethiopia [7], 95 % of confidence interval, 5 % marginal error, adding 15 % for non-response rate the final sample size was 308.

### Sampling technique and procedures

A consecutive sampling technique was employed among mother-newborn pairs in the study area. For the qualitative aspect of the study, members of the FGD were selected purposively.

### Study variables

The tool was designed to have four part that include socio-demographic characteristics (age, religion, ethnicity), obstetric health (Chronic illness of the mother, history of abortion, use of family planning, etc.) anthropometric measurements (Maternal weight, Height, MUAC, weight of the newborn etc.), socio-cultural patterns of feeding, (food taboos, food preference, fasting during pregnancy).

### Data collection instruments

Quantitative: The data were collected using a structured pre-tested interviewer guided questionnaire which was prepared by reviewing similar literatures. Six data collectors who were midwives (i.e. two from each hospital) that can speak the local language (Tigrigna Language), were recruited and two supervisors were selected from each woreda Health Office according to their research experiences.

Qualitative: In order to explore the socio-cultural pattern of the community a total of three focus group discussions (FGD) which consists of 7-8 individuals in each group (i.e. one FGDs in each hospital), was conducted using semi-structured, open-ended questionnaires in order to provide more insight in to the culture of feeding of pregnant mothers in the study area. The members of each FGD were selected purposively by the supervisors and the principal investigator and moderated by principal investigator with the assistance of trained note taker and tape recorder.

### Data quality control

To assure the quality of the data properly designed data collection instrument and training of data collectors were done. The enumerators and the supervisor were given training for two days on procedures, techniques and ways of collecting the data including calculating gestational age, measuring weight, height and MUAC. The collected data were reviewed and checked for completeness by principal investigator & supervisors each day. The questionnaire was initially prepared in English and then translated in to Tigrigna. The Tigrigna version was again translated back to English to check for any inconsistencies or distortions in the meaning of words & concepts. The questionnaire was pre tested prior to the actual data collection on 15 respondents that were not included in the main survey. The babies were weighed on beam balance up to 20 g accuracy within 2 hrs of delivery and all mothers were interviewed and examined within 6 hrs of delivery. The measurements were made on the participants wearing a minimum amount of clothing. The weights of pregnant women were recorded at the early first trimester during their first visit and continued in every visit by using a beam balance with an accuracy of 100 g. A standard WHO Mid upper arm circumference (MUAC) measuring tape was used to measure maternal mid upper arm circumference. The weight gain during pregnancy was estimated by subtracting the early first trimester weight from the last measured weight before delivery. Height was measured in cm using calibrated measuring steel attached to the beam balance. The pregnant women were asked to maintain an upright and erect posture with her feet together a horizontal headpiece was lowered onto the women’s head. Chronic medical illness has been operationalized as having any of the following illnesses: hypertension, diabetes mellitus, congestive heart failure, HIV during the course of current pregnancy.

### Ethical considerations

Formal letter of permission was obtained from Bahir Dar University ethical committee and Tigray Regional health bureau. The respondents were informed about the objective and purpose of the study and verbal consent was obtained from each respondent during data collection.

### Data management and analysis

Data were entered and analyzed using SPSS version 16.0 computer software package. Bivariate analysis was done with 95 % confidence interval to infer association, controlling the effect of confounders using multivariate logistic regression model. Variable which were found statistically significant at *p* < *0.05* identified as independent predictors of LBW. The Hosmer-Lemeshow goodness of fit test provides an evidence for model fit with the predictor (*p* = *0.415*). Multicollinearity effect has been checked in the multivariate model and all the variables were having a standard error <2.0. In regard to qualitative section of this study, the tape-recorded and the notes taken during Focus group discussion (FGD) were transcribed and then translated from Tigrigna to English in verbatim way. After the interviews listened for many times, the transcripts were reduced, coded and categorized in to themes, and finally triangulated with the quantitative results.

## Results

### Socio-demographic characteristics of the respondents

The study included 308 mothers/newborn pairs who delivered in governmental hospitals of central zone of Tigray, Northern Ethiopia; having a response rate of 100 %. The mean age of the study participants was 26.90 years (SD ± 5.86). Out of the respondents majority 254 (82.5 %) mothers were between 18-34 years of age. Two hundred ninety eight (96.8 %) of the respondents were married, 85 (27.6 %) mothers are unable to read and write and less than ten percent 24 (7.8 %) mothers did complete college and above. Related with the religion of respondents 295 (96.1 %) were Orthodox Christians (Table 1).

**Table 1:** Socio-demographic characteristics of study participants (n = 308)

Variables	Categories	Frequency	Percentage (%)
Age (in yrs)	<18 years	9	2.9
	18-34 years	254	82.5
	> = 35 years	45	14.6
Religion	Orthodox Christian	296	96.1
	Muslim	12	3.9
Educational status	Unable to read & write	85	27.6
	Able to read and write	67	21.8
	Primary school (1-8)	56	18.2
	Secondary school (9-12)	76	24.7
	College and above	24	7.8
Ethnicity	Tigray	303	98.4
	Amhara	5	1.6
Residence	Urban	179	58.1
	Rural	129	41.9

### Obstetric health characteristics of the study participants

One hundred seventy nine (58.1 %) of the mothers had history of contraceptives use. The type of pregnancy that the mothers had, wanted and planed accounts for 274 (89 %), the unwanted and unplanned category accounts 16 (5.2 %). Concerning to inter-pregnancy interval, 268 (87 %) were delivered more than two years ahead of the index pregnancy. Almost all participants 303 (99 %) had history of antenatal care (ANC) follow up, of whom 215 (69 %) had 2-4 follow up and 57 (18.5 %) had greater than four visits during the current pregnancy. A significant number of mothers (92.5 %) have received dietary counseling during the ANC follow up (Table 2).

**Table 2:** Obstetric health characteristics of study participants, Aug 2013

Variable	Category	Frequency	Percentage (%)
History of contraceptive use	Yes	179	58.1
	No	129	41.9
Complication during pregnancy	Yes	34	11
	No	274	89
ANC attendance of the mother	Yes	305	99
	No	3	1
Pregnancy type	Wanted and planed	274	89
	Wanted but unplanned	18	5.8
	Unwanted & unplanned	16	5.2
Number of ANC visit (n = 305)	<2	36	11.7
	2-4	215	69.8
	>4	57	18.5
History of abortion	Yes	58	18.8
	No	250	81.2
Pregnancy interval in years	<2 years	40	13
	> = 2 years	268	87
	>6	29	9.4
Dietary counselling (n = 305)	Yes	285	92.5
	No	23	7.5
Chronic medical illnesses	Yes	11	3.6
	No	297	96.4

With regard to the birth weight of the newborns 263 (85.4 %) were above or equals to 2500 gs and the weight of children less than 2500 gs were 45 (14.6 %). The mean and standard deviations of the birth weights were 3094.9 ± 587.6 grams. Among the total respondents, 113 (36.7 %) had less than 50 kg of weight (Table 2). Mothers who were > =150 cm in height accounts for 280 (90.9 %) whereas minority 28 (9.1 %) mothers were <150 cm in height (Table 3).

**Table 3:** Anthropometric profiles of the study participants, central Tigray, Aug 2013

Variables	Categories	Frequency	Percentage (%)
Height of the mother	<150 cms	28	9.1
	> = 150 cms	280	90.9
Weight of the mother	<50 kgs	113	36.7
	> = 50 kgs	195	63.3
MUAC of the mother	<21 cms	109	35.4
	> = 21 cms	199	64.6
Weight of the newborn	> = 2500 gms	263	85.4
	<2500 gs	45	14.6
Gestational age	<37 weeks	14	4.5
	> = 37 weeks	295	95.5

### Logistic regression analysis of maternal factors associated with LBW

In the multivariate logistic regression analysis model, controlling potential confounders maternal place of residence, gestational age, weight of mother and presence of chronic medical illnesses were found to be significant predictor of low birth weight.

Residence of the mother was strongly associated with low birth weight, mothers residing in rural area were more than 4 times more likely to have LBW babies when compared to those mothers who live in urban (AOR = 4.34 (95 % CI = 1.98, 9.48)). The risk of having LBW baby was more than two folds higher in mothers who had a body weight less than 50 kg when compared to mothers having body weight > = 50 kg (AOR = 2.23 (95 % CI = 1.06, 4.80)).

Gestational age of the fetus on the risk of having low birth weight babies, the odds of being LBW in babies born before gestational age of 37 weeks was 18 times higher when compared to babies born at gestational age of 37 weeks and more (AOR = 18.52 (95 % CI = 4.94, 69.43)).

Of the maternal obstetric factors the presence of any chronic medical illnesses during current pregnancy was assessed and it was found to be associated significantly with LBW. The odds of being LBW in babies born from mothers with history of chronic medical illnesses during their current pregnancy were found to have greater than five times chance of delivering LBW baby when compared with mothers with no history of chronic medical illnesses (AOR = 5.33 (95 % CI = 1.12, 25.45)) (Table 4).

**Table 4:** Maternal factors associated with low birth weight from logistic regression analysis, central Tigray, Ethiopia, Aug 2013

Variables	Categories	LBW		COR (95 %, CI)	AOR (95 %, CI)
		Yes (%)	No (%)		
Height of mother (in cm)	<150 cm	8 (28.6)	20 (71.4)	2.62 (1.08, 2.63)	3.02 (0.90, 10.10)
	> = 150 cm	37 (13.2)	243 (86.8)	1.00	1.00
Nutritional counseling	Yes	37 (13.0)	248 (87)	1.00	1.00
	No	8 (34.8)	15 (65.2)	3.575 (9.02, 1.42)	0.97 (0.27, 3.51)
Residence	Urban	16 (8.9)	163 (91.1)	1.00	1.00
	Rural	29 (22.5)	100 (77.5)	2.954 (1.53, 5.71)	4.34 (1.99, 9.48)**
History of FP use	Yes	28 (15.6)	151 (84.4)	1.222 (0.64, 2.34)	1.47 (0.68, 3.19)
	No	17 (13.2)	112 (86.8)	1.00	1.00
Presence of chronic medical illnesses	Yes	3 (27.3)	8 (72.7)	2.277 (0.58, 8.93)	5.3 (1.12, 25.45)**
	No	42 (14.1)	255 (85.9)	1.00	1.00
Weight of the mother (in kg)	<50 kg	24 (21.2)	89 (78.8)	2.234 (1.17, 4.23)	2.26 (1.06, 4.80)**
	> = 50 kg	21 (10.8)	174 (89.2)	1.00	1.00
MUAC of mother (in cm)	<21 cms	20 (18.3)	89 (81.7)	1.564 (0.82, 2.97)	1.02 (0.43, 2.41)
	> = 21 cms	25 (12.6)	174 (87.4)	1.00	1.00
Gestational age (in wks)	<37 weeks	10 (71.4)	4 (28.6)	18.5 (5.50, 62.16)	18.5 (4.94, 69.4)**
	> = 37 weeks	35 (11.9)	259 (88.1)	1.00	1.00
History of abortion	Yes	12 (20.7)	46 (79.3)	1.71 (0.82, 3.57)	1.54 (0.63, 3.79)
	No	33 (13.2)	217 (86.8)	1.00	1.00

N.B. 1 = Reference Category, ** = Significant at p-value <0.05 in multivariate analysis

### Qualitative findings

The socio-demographic characteristics of the discussants was, 7 mothers (34 %) in the FGD were from rural and 3 (14 %) of the participants were Muslim followers, and the rest 18 (86 %) were Christian and the urban dwellers account for 14 (66 %) their ages ranged from 19-45 yrs.

Three identified central themes under which the rest were included: Culturally prohibited foods and reasons of prohibition during pregnancy, feeding preferences and culturally recommended foods for pregnant mothers and fasting beliefs and practices during pregnancy and child birth.

#### Culturally prohibited foods and reasons of prohibition during pregnancy

Majority of respondents described that the presence culturally prohibited food items and their practices have the tendency to affect their feeding patterns during the course of pregnancy. It is considered as refusals if they are not carried out as prescribed by their ancestors. This was supported by many of the respondent when they recited their practice and tradition of feeding.

“*Food items like that of meat*, *fat*, *and butter are not encouraged for a pregnant mothers to eat frequently, because these food types leads the fetus to grow larger beyond the birth canal could tolerate and lead to severe complications during child birth”* 33 yr old mother multi-para.

Another discussant added on this position as follows:

“…*in addition to animal products honey shouldn’t be eaten during pregnancy because it leads to a pain full false labor that persists longer and it is also the main cause of constipation during the course of pregnancy.*” 39 years old multi-para mother

Similarly an FGD participant also disclosed her fear as follows:

*“I was afraid of eating eggs, meat, and much cow’s milk while pregnant and I used to replace them with vegetables and some cereals products, this is because my parents advise on my feeding sequence to avoid the unnecessary growth of the fetus in my womb…”* A 21 yrs old primi mother

#### Culturally recommended foods and feeding preferences of pregnant mothers

On the issue of food types which were considered safe and right for a pregnant woman during the course of pregnancy and child birth were almost supported by the study participants.

*“I used to prefer eating Injera (Ethiopian staple food), bread and vegetables, infrequently milk and animal products while I was pregnant, but soon shifted to porridges and frequent feeding of animal products after birth because I was told that animal products are not good during pregnancy to consume frequently”* A 32 years old multi-para

A 29 years old woman claimed: *“…food items that are considered preferable based on advice of elders during pregnancy generally are all kinds of cereals their products like wheat flour and vegetables especially those that are eaten cooked”.*

A 42 years old and mother of 5 children added *“Frequent consumption of linseed in the late weeks of pregnancy is helpful in softening and lubricating the body of expectant mother, there by facilitate the course of child birth”*

On the other hand respondents were seen to face difficulties on which food stuffs to consume during pregnancy regarding the sources of information at hand.

*“We have strong social ties in every aspect of life and it is difficult to ignore the advice of our elder family members & neighbors regarding the diet during pregnancy, we still obey their experiences since it could have truth in some instances and I sometimes strictly follow it despite the advice of health professionals and I always felt frightened to take much animal products” A* 26 years old mother of two children

Other discussants said the traditionally recommended foods were more accepted in the community many respondents listed them among the local foods to be similar as above. Financial concerns were common among participants; especially those who were unemployed that explain the preference of food depends on their income.

#### Fasting beliefs and practices during pregnancy and child birth

The respondents view regarding fasting during pregnancy was also incorporated in this study, there are few contradictions among the discussants on postponement of fasting after delivery. A 43 years old multi-para said *“I was not fasting all the fasting hours during the course of my pregnancy but I never took non-fasting foods like meat, eggs and milk during the fasting time because I do not need to breach the rules of my religion and isolate myself from my family for this reason…”*

Women participating in the FGD frequently relied on parents, relatives and religious rules for support during pregnancy. Participants were asked for their perception what if they did not fast. “*Older parents and religious rules will never accept you to eat non-fasting foods whatever your pregnancy status rather they strongly advocate to fasting and have strong link with church, you did not obey this it is considered as a sinful act…”*

Another discussant added similar outlook “…*If someone disobeys the religious rules(if a woman is not fasting) either the mother will face problems during child birth or the child will be born with ill health that’s why I prefer to stick with it because I do not want to have unhealthy baby”.*

The issue of fasting among Christian followers is a bit different, that the majority of the discussants did not accept to taking non-fasting foods during pregnancy. A 41 years old woman claimed “…*I will never ask my religious father to such a permission (to having non-fasting foods) while pregnant because such permissions are allowed for conditions beyond your capability and for seriously ill individuals*”.

Generally, healthy and varied diet is important during pregnancy and the diet must provide sufficient energy and nutrients to meet the maternal daily requirements & the growing fetus. Pregnant women should try to consume plenty of iron- and folate-rich foods, and Vit.D throughout pregnancy, the important fatty acids and amino acids are crucial for fetal development of the brain, nervous system and retina [9] that found in plenty in animal products but these nutrients are prohibited culturally in the society.

## Discussion

In this study an attempt has made to determine the prevalence and associated factors of low birth weight in the study area. Results of this study revealed that maternal weight, gestational age, residence, and presence of chronic medical illness were found to be significant predictors for low birth weight. The prevalence of low birth weight was found to be 14.6 %, which is similar with the LBW levels of sub-Saharan Africa countries [1] and it is less than the prevalence of LBW found in some parts of the country (17.1 %, 22.5 %) observed in Gondar and Jimma University hospitals respectively [5, 7]. This difference might be due to the previous studies were carried out in specialized hospitals where many of the pregnant women were referred from peripheral hospitals because of high risk pregnancy. The risk having LBW was more than two fold for women weighing less than 50 kg compared to mothers with greater than or equals to 50 kg body weight. This is in line with the study conducted in India and Pakistan [10–12]. This could be due the fact that maternal weight less than 50 kg might be related with poor maternal malnutrition during infancy and child hood periods that persist to affect the new offspring. Presence of chronic medical illness during pregnancy was found to be independently associated with LBW. The odds of having LBW baby in mothers with history of chronic medical illnesses was found to be greater than five folds comparing to mothers with no history of chronic medical illnesses. This result is consistent with the study conducted in Malaysia and WHO findings in many sub countries [13, 14]. This could be due to lack of medical checkups of chronic illnesses strictly during pregnancy that may go unrecognized leading to negative outcomes on the fetus in the womb. Gestational age was also independently associated with the occurrence of low birth weight; the likelihood of having LBW in babies born before gestational age of 37 weeks was 18 times higher compared to that of babies born at gestational age of 37 weeks and above. This result is in line with studies conducted in Addis Ababa [6] and WHO rating in different countries [3]. The reason for this is well recognized that as the gestational age of the fetus falls below the acceptable range of time, the body weight of the fetus falls dramatically due to prematurity.

The place of residence of the mothers was independently associated with the occurrence of low birth weight. Mothers residing in rural area were greater than 4 times more likely to have LBW babies when compared to those mothers who live in urban. This is consistent with the study done in Jimma zone and Tanzania [7, 12] this could be due to the accessibility of medical services, health information, and nutritional awareness which are more prominent as the woman resides in urban area than rural area. In addition rural residents do not appear to health institution at greater risk of poor perinatal outcome than their urban counterparts.

The qualitative part of this study design depicted that the culture of preference of nutritionally poor food items during pregnancy; it was profound and largely performed based on their family, parents and religious leaders despite medical advice. This predisposes mothers to miss timing nutritious food items and malnutrition during conception. The condition of malnutrition is also supported by the quantitative results of this study that showed the presence of maternal malnutrition as recognized on the anthropometric measurements that larger numbers of mothers were unable to achieve the average MUAC of pregnant women and weight during pregnancy.

### Strengths of the study

The use of qualitative study design to explore the effects of socio-cultural variables in this study.

The involvement of midwives during anthropometric measurements during the survey and medical records were cross checked to confirm important variables such as patients’ hypertensive status, obstetric history, and antenatal history.

### Limitations of this study

Inability to include mothers who deliver at home and the presence of selection bias.

A recall bias may be introduced during data collection time as some of the variables need a recall to situations happened few weeks/months back to the actual data collection time.

## Conclusion

This study depicted that the prevalence of LBW was found to be higher, pertaining the maternal factors associated with LBW, it has concluded LBW was found to be affected by rural place of residence, presence of any chronic medical illnesses, preterm birth (gestational age <37 wks) and maternal weight <50 kg. The qualitative part of this study found a strong influence of the socio-cultural beliefs in the nutritional intake of pregnant women. It particularly suggested that the presence of prejudice on animal products for fear of complications related to large baby during child birth. Preference nutritionally poor food items and miss timing of nutritious foods during pregnancy and child birth was also prominent accentuating the occurrence of low birth weight.

Based on the findings of this study the following recommendations are forwarded:

Attendants of ANC should receive disease specific counseling by skilled health personnel with emphasis given to mothers with chronic medical illnesses. Policy makers and health planers should optimize programs of community based nutritional promotion programs giving due attention to rural segment of the community. The zonal and woreda health offices should work on the adequacy of nutritional counseling given to expectant mothers with due emphasis given to mothers lower than 50 kg in weight. The tradition of feeding of pregnant mothers was determined by paramount influence of religious leaders & the community as a whole therefore, there is a need to intensively discussions on the issue of prohibited foods with regional health bureau to averting this problem. Community based further studies are also needed to identify the effects of seasonal variations of nutrition on the effects of birth outcome.

## References

[1] United Nations Children’s Fund and WHO: Low Birth Weight country, regional and global estimates. New York; 2004*.*available from [http://www.unicef.org/publications/index_24840.html]. Last accessed 24 May 2013

[2] Taylor HG, Minich NM, Klein N, Hack M (2004). Longitudinal outcomes of very low birth weight: Neuropsychological findings. J Int Neuropsycho Soc.

[3] Siza JE (2008). Risk factors associated with low birth weight of neonates among pregnant women attending a referral hospital in northern. Tanzan J Health Res.

[4] Rajaeefard A, Mohammadi M, Choobineh A (2007). Preterm delivery risk factor: a prevention strategy in Shiraz, Islamic Republic of Iran. East Mediterr Health J.

[5] Megabiaw BM, Zelalem M, Mohammed N (2012). Incidence and correlates of low birth weight at a referral hospital in Northwest Ethiopia. Pan Afr Med J.

[6] Fikre E, Aklilu M (2000). Change in birth-weight of Hospital-Delivered neonate in Addis Ababa, Ethiopia. J Health Dev.

[7] Tema T (2006). Prevalence and determinants of LBW in Jimma zone, Southwest Ethiopia. East Afr Med J.

[8] The 2011 Ethiopian Demographic and Health Survey: Report. Addis Ababa; 2011

[9] Williamson S (2006). British Nut Foundation Nut Bul.

[10] Mumbare SS, Maindarkar G, Darade R, Yenge S, Tolani MK (2012). Maternal risk factors associated with term low birth weight neonates. Indian Pediatrics J.

[11] Aurora S, Vishnu BB, Habibullah S (1994). Maternal nutrition and birth weight. Indian J Mat Child Hlth.

[12] Edris M, Erakli G (1996). The prevalence of LBW and factors associated with LBW in Gondar, North west Ethiopia. Ethiop J Health Dev.

[13] Hematram Yadav MBA (2013). Maternal Factors in Predicting Low Birth Weight Babies. Med J Malaysia.

[14] Kramer MS (1987). Determinants of Low Birth Weight: Methodological assessment and meta-analysis. Bulletin The WHO.

